# Postoperative Neck Ultrasonography Surveillance After Thyroidectomy in Patients With Medullary Thyroid Carcinoma: A Multicenter Study

**DOI:** 10.3389/fendo.2018.00102

**Published:** 2018-03-15

**Authors:** Hye Shin Ahn, Dong Wook Kim, Yoo Jin Lee, Chang Yoon Lee, Ji-hoon Kim, Yoon Jung Choi, Song Lee, Inseon Ryoo, Jung Yin Huh, Jin Yong Sung, Jin Young Kwak, Hye Jin Baek

**Affiliations:** ^1^Department of Radiology, Chung-Ang University Hospital, Chung-Ang University College of Medicine, Seoul, South Korea; ^2^Department of Radiology, Busan Paik Hospital, Inje University College of Medicine, Busan, South Korea; ^3^Department of Radiology, Research Institute and Hospital, National Cancer Center, Gyeonggi, South Korea; ^4^Department of Radiology, Seoul National University Hospital, Seoul National University College of Medicine, Seoul, South Korea; ^5^Department of Radiology, Kangbuk Samsung Hospital, Sungkyunkwan University School of Medicine, Seoul, South Korea; ^6^Department of Radiology, Chak Han Madi Hospital, Incheon, South Korea; ^7^Department of Radiology, Korea University Guro Hospital, Korea University College of Medicine, Seoul, South Korea; ^8^Department of Radiology and Research Institute of Radiology, University of Ulsan College of Medicine, Asan Medical Center, Seoul, South Korea; ^9^Department of Radiology, Thyroid Center, Daerim St. Mary’s Hospital, Seoul, South Korea; ^10^Department of Radiology, Severance Hospital, Research Institute of Radiological Science, Yonsei University College of Medicine, Seoul, South Korea; ^11^Department of Radiology, Gyeongsang National University School of Medicine, Gyeongsang National University Changwon Hospital, Changwon, South Korea

**Keywords:** thyroid, malignancy, medullary thyroid carcinoma, ultrasonography, recurrence, surveillance

## Abstract

**Background:**

For detecting tumor recurrence of medullary thyroid carcinoma (MTC) in the neck, an appropriate frequency and interval of postoperative ultrasonography (US) surveillance remains unclear. This study aimed to assess an appropriate interval and frequency of postoperative neck US surveillance for detecting tumor recurrence in patients who had undergone thyroid surgery due to MTC.

**Methods:**

A total of 86 patients who had undergone thyroid surgery for the treatment of MTC and had at least one postoperative US follow-up examination at any of nine affiliated hospitals were included. Postoperative follow-up US, clinical, and histopathological results of patients were reviewed. The tumor recurrence/persistence rate of MTC was investigated, and the interval and session number of postoperative follow-up US and clinicopathologic factors were compared between tumor recurrence/persistence and non-recurrence groups.

**Results:**

Of the 86 patients, 22 (25.6%) showed tumor recurrence/persistence. Of the 22 patients with tumor recurrence/persistence, 11 (50%) showed structural recurrence/persistence in the neck on follow-up US. In these 11 patients, the mean interval and session number of postoperative follow-up US between initial surgery and the first US detection of recurrence/persistence was 41.3 ± 39.3 months (range, 6–128 months) and 2.6 ± 2.3 (range, 1–8), respectively. On follow-up US, 6 (54.5%, 6/11) were diagnosed with tumor recurrence/persistence within 3 years of the initial surgery. Tumor recurrence/persistence was significantly correlated with TNM stage (*p* < 0.001) and multiplicity/bilaterality (*p* = 0.013).

**Conclusion:**

For detecting MTC recurrence/persistence, postoperative US surveillance at 1-year intervals may be sufficient within the first 3 years after thyroid surgery, but depending on the presence of relevant risk factors, annual or biannual US surveillance may be recommendable for 4–10 years after thyroid surgery.

## Introduction

Medullary thyroid carcinoma (MTC) is an uncommon thyroid cancer which accounts for 3–10% of all thyroid cancers (1, 2). MTCs are derived from the parafollicular C-cells that produce calcitonin, and serum calcitonin level is known to be a sensitive and specific marker for MTC (3). However, several reports suggest that advanced MTC may dedifferentiate with a subsequent decrease in calcitonin production (4, 5).

The prognosis of MTC is intermediate between differentiated and anaplastic thyroid carcinomas (1). The natural course of MTC varies from very slow progression or stable disease extending over decades to rapid progression and survival of just a few years (1). The reported 5-year survival of MTC is 78–91%, and the reported 10-year survival is 61–88% (1, 6). Distant metastasis, which is reportedly observed at initial presentation in 7–23% of patients, is known to be the main cause of MTC-related mortality (7). Distant metastasis, older age at diagnosis, and more advanced primary tumor or nodal stage have been proposed as prognostic factors for adverse outcomes (8, 9). Also, serum kinetics of MTC markers, such as calcitonin and carcinoembryonic antigen, may be alternative predictors of survival (10).

Thyroid ultrasonography (US) has been used to differentiate between malignant and benign thyroid nodules, to guide fine-needle aspiration of suspicious thyroid nodules, and to detect tumor recurrence after thyroid surgery (11, 12). Due to an increase in thyroid US screening, the detection rate of small thyroid cancers has increased substantially, and earlier detection of smaller MTCs is possible (13–15). In particular, Elisei et al. reported US follow-up results including the neck in patients who had undergone thyroid surgery due to MTC (16). They recommended that postoperative neck US be undertaken at interval of 6–12 months. However, this may be too short interval, and can result in unnecessary medical costs. To the best of our knowledge, an appropriate interval for postoperative US surveillance remains unclear. This study aimed to determine the detection rate of tumor recurrence/persistence and characteristics of postoperative follow-up US in MTC patients by analyzing follow-up US results in patients who had undergone thyroid surgery due to MTC.

## Materials and Methods

### Patients

This retrospective analysis was based on patient data collected from nine university-affiliated hospitals. The institutional review boards of all participating institutions approved the study, and the need for informed consent was waived due to the retrospective nature of the study. From January 2005 to April 2011, only patients who met the following criteria were consecutively included, although the start time for patient selection differed among the institutions due to the scarcity and variations in the number of MTC cases at each institution: (1) patients who had undergone thyroid surgery for the treatment of MTC, (2) patients who had at least one postoperative US follow-up examination during the follow-up period, (3) patients who had been clinical followed-up for more than 5 years after the initial thyroid surgery, and (4) patients in which the presence or absence of tumor recurrence/persistence could be determined. Ultimately, a total of 86 patients (53 women and 33 men, age range 7–76 years, mean age 53.1 ± 11.8 years) were included in the study.

### Neck US Examinations and Follow-ups

All US examinations were performed using one of the following high-resolution US systems: (1) iU 22 (Philips Medical Systems, Bothell, WA, USA), (2) HDI 5000 (Philips Medical Systems, Bothell, WA, USA), (3) LOGIQ9 (GE Healthcare, Milwaukee, WI, USA), (4) Aplio SSA-770A (Toshiba Medical Systems, Tokyo, Japan), and (5) EUB-7500 (Hitachi Medical Corporation, Tokyo, Japan). A 5–12-MHz or an 8–15-MHz linear-array transducer was used. Faculty radiologists specializing in head-and-neck imaging or supervised board-certified radiologist who were participating in head-and-neck radiology fellowship training performed all US examinations. Postoperative US follow-up in MTC patients was commonly performed at 0.5-, 1-, or 2-year intervals at each institution. The following features were investigated *via* US examination: suspicious thyroid nodules in the remnant thyroid gland, masses in the postoperative thyroid bed and perithyroidal neck area, and suspicious lymph nodes or masses in the neck. In all patients, the interval (i.e., period between the initial surgery and postoperative follow-up US examination) and session number of postoperative follow-up US were investigated. In the tumor recurrence/persistence group, the interval and session number of postoperative neck US where tumor recurrence/persistence was first detected were recorded.

### Thyroid Surgery and Histopathology

Thyroid surgery was performed by board-certified surgeons of the affiliated hospitals. The extent of thyroid surgery was determined by multiple factors, including patient age, family history, tumor size, extrathyroidal tumor extension, multifocality, bilaterality, nodal metastasis, and distant metastasis. In the histopathological analysis, the intraglandular location of the MTC, perithyroidal tumor invasion within or beyond surgical margins, nodal metastasis, and the presence of satellite MTC were recorded.

### Classification of Tumor Recurrence/Persistence and Non-Recurrence

Nine board-certified radiologists from each of the affiliated hospitals retrospectively reviewed the postoperative follow-up US, clinical, and histopathological results of the patients included in the study. The size, location, multiplicity, and TNM stage of tumors were investigated on the basis of US images and histopathological results. TNM stage was based on the guidelines of the American Joint Committee on Cancer (17). The sites of recurrence/persistence were classified as nodal, non-nodal, or unknown. Non-nodal recurrence/persistence was defined as the presence of an MTC in the remnant thyroid gland, postoperative thyroid bed, or perithyroidal neck area during the follow-up period after surgery as determined *via* histopathological examinations. Nodal recurrence/persistence was defined as the presence of a metastatic lymph node from MTC in the neck area during the follow-up period after surgery. US features of metastatic lymph nodes and local recurrent masses in the operative bed were characterized in accordance with previous reports (18, 19). In patients with tumor recurrence/persistence, the location, diagnostic method, and management of tumor recurrence/persistence were reviewed. An interval and session number of postoperative follow-up US were also recorded.

Serum calcitonin levels at the time of initial preoperative laboratory investigations and final serologic tests were investigated. In the patients with tumor recurrence/persistence, the serum calcitonin level at the time of diagnosis of recurrence/persistence was defined as the final serum calcitonin value. Serum calcitonin levels were measured using immunoradiometric assays in eight affiliated hospitals, and chemiluminescence immunoassays in one hospital. The cutoffs for calcitonin levels were 0–10 pg/mL in seven of the hospitals using immunoradiometric assays, 0–14 pg/mL in the remaining one hospital using immunoradiometric assays, and 0–8.4 pg/mL (male) or 0–5.0 pg/mL (female) in the one hospital using chemiluminescent immunoassays. In previous reports, a normal basal calcitonin level of <10 pg/mL has been used to exclude remnant MTC, and ≥10 pg/mL of serum calcitonin has been deemed indicative of recurrence/persistence (20, 21).

Based on the literature, postoperative disease status within 6 months of surgery and at the last follow-up were classified as follows: (1) no tumor recurrence/persistence (biochemical remission as defined by a serum calcitonin level of <10 pg/mL and no structural disease evident *via* neck US or other available imaging modalities during postoperative follow-up), (2) only biochemical recurrence/persistence (serum calcitonin level of ≥10 pg/mL with no evidence of structural disease during postoperative follow-up), and (3) structural recurrence/persistence (with identification of structural recurrence/persistence on neck US or other available imaging modalities during postoperative follow-up) (20–22).

### Statistical Analysis

All statistical analyses were performed using SPSS for Windows (version 21.0, SPSS, Chicago, IL, USA). Categorical variables were compared using the chi-square test or Fisher’s exact test, and continuous variables (age, tumor size, and serum calcitonin level) were compared using Student’s *t*-test. A *p* <0.05 was considered to indicate statistical significance. The prevalence of MTC recurrence/persistence was calculated, and the interval and session number of postoperative follow-up US were compared between the recurrence/persistence and non-recurrence groups. Also, we assessed correlations between tumor recurrence/persistence and variable clinicopathological factors, to predict prognosis.

## Results

Of the 86 patients, the mean size of the primary MTC was 16.9 ± 13.7 mm (range 3.0–88.6 mm). The locations of the primary MTC included the right lobe (*n* = 59), the left lobe (*n* = 26), and the isthmus (*n* = 1). Thyroid surgery was performed in all 86 patients, and the TNM stages based on histopathology of the surgical specimens are shown in Table 1. More than 50% of patients were diagnosed with intraglandular MTC. There were no cases of tumor invasion into the adjacent tracheal or esophageal wall. Nodal metastases in the lateral neck were demonstrated in 25 patients (29.1%). Distant metastasis was only noted in one patient (1.2%). The interval between thyroid surgery and the last follow-up US was 60 or more months in 74 patients (86.0%) and 90 or more months in 22 patients (25.6%).

**Table 1:** Demographics and characteristics in the 86 patients.

Items	
Age (mean ± SD, years)	53.1 ± 11.8 (range, 7–76)
Sex	Female: male = 53 (61.6): 33 (38.4)
**Type of thyroid surgery**	
Total thyroidectomy	78 (90.7)
Hemithyroidectomy	7 (8.1)
Subtotal thyroidectomy	1 (1.2)
Size of primary MTC (mean ± SD, mm)	16.9 ± 13.7 (range, 3–88.6)
**Location of primary MTC**	
Right	59 (68.6)
Left	26 (30.2)
Isthmus	1 (1.2)
**T stage**	
T1a	22 (25.6)
T1b	29 (33.7)
T2	10 (11.6)
T3	25 (29.1)
T4a	0
**N stage**	
Nx (unknown)	3 (3.5)
N0	38 (44.2)
N1a	20 (23.3)
N1b	25 (29.1)
**M stage**	
Mx (unknown)	15 (17.4)
M0	70 (81.4)
M1	1 (1.2)
**Multiplicity/bilaterality**	
Solitary	70 (81.4)
Satellite MTC in the ipsilateral lobe	6 (7.0)
Satellite MTC in the contralateral lobe	1 (1.2)
Satellite MTC in both lobes	9 (10.5)
**Interval of the last follow-up US (mean ± SD, months)**	78.6 ± 24.9 (range, 23–141)
**Session number of the last follow-up US**	7.0 ± 3.3 (range, 1–18)

*Data presented in parentheses are percentage of each item*.

*MTC, medullary thyroid carcinoma*.

**Figure 1:**
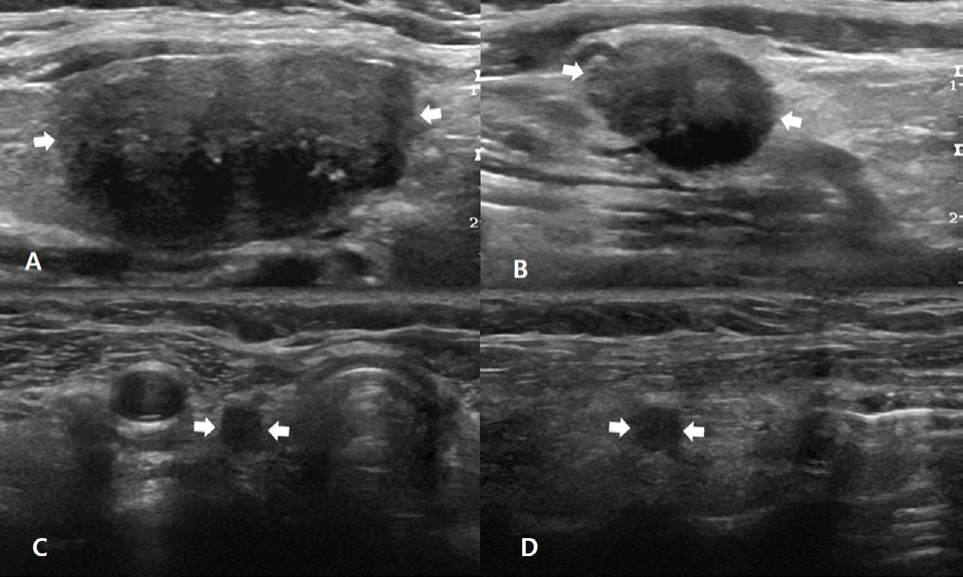
A 51-year-old man with non-nodal recurrence/persistence of medullary thyroid carcinoma (MTC). Preoperative longitudinal gray-scale sonograms show a primary MTC (arrows, 25.0 mm at its largest diameter) in the right thyroid lobe **(A)**, and a metastatic lymph node (arrows) in the right lateral neck **(B)**. On histopathological examination after total thyroidectomy with lateral neck dissection, there was perithyroidal tumor invasion (T3) and nodal metastasis in the right neck (N1b). The patient exhibited biochemical remission after thyroid surgery, and there was no focal lesion in the postoperative thyroid beds and necks on 6, 12, 18, or 24-month follow-up ultrasonography (US) after thyroid surgery. In the fifth follow-up US at 30 months after thyroid surgery, transverse **(C)** and longitudinal **(D)** gray-scale sonograms showed an oval hypoechoic nodule (arrows, 8.0 mm at its largest diameter) in the right postoperative thyroid bed. After US-guided fine-needle aspiration for this lesion, cytology revealed suspicious MTC, and this lesion was confirmed as non-nodal recurrence/persistence of MTC after the second-look surgery.

**Figure 2:**
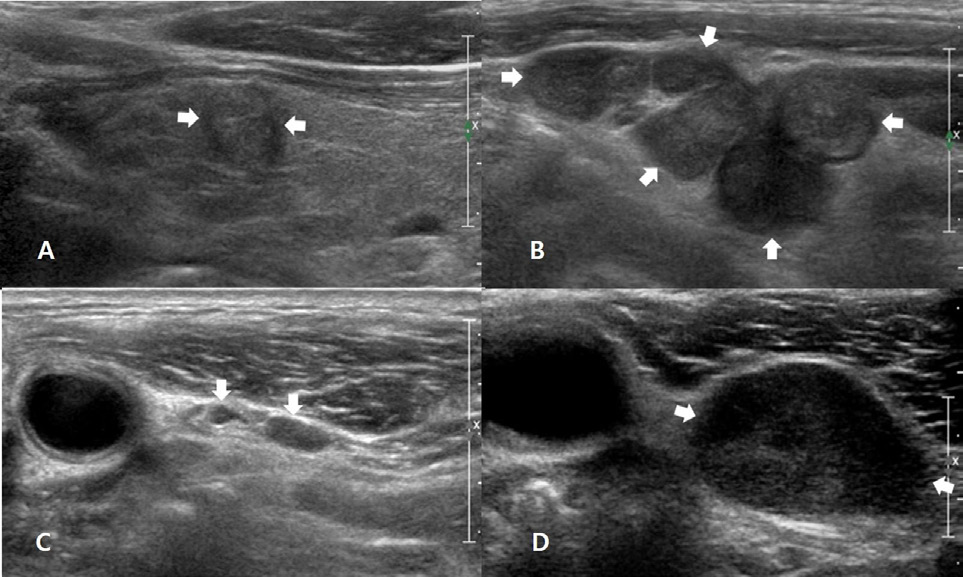
A 54-year-old man with nodal recurrence/persistence of medullary thyroid carcinoma (MTC). Preoperative longitudinal gray-scale sonogram shows an MTC (arrows, 9.3 mm at its largest diameter) in the left thyroid lobe **(A)** and multiple metastatic lymph nodes (arrows) in the left lateral neck **(B)**. On histopathological examination after total thyroidectomy with lateral neck dissection, there was perithyroidal tumor invasion (T3) and nodal metastasis in the left neck (N1b). The patient showed biochemical remission after thyroid surgery, and the first follow-up ultrasonography (US) at 12 months after thyroid surgery showed normal lymph nodes (arrows) in the left level-III neck **(C)**. On the second follow-up US at 25 months after thyroid surgery, a transverse gray-scale sonogram **(D)** shows a suspicious LN (arrows, 19.9 mm at its largest diameter) in the left level-III neck. After US-guided fine-needle aspiration for this node, cytology revealed nodal metastasis of MTC, and this lesion was confirmed after the second-look surgery.

Of the 86 patients, 64 (74.4%) exhibited no tumor recurrence/persistence during the follow-up period. Of the 22 (25.6%) remaining patients, 11 (50%) showed biochemical recurrence/persistence with no structural recurrence/persistence and 11 (50%) showed structurally identified recurrence/persistence (Figures 1 and 2).

Of the 11 patients with structural recurrence/persistence, tumor recurrence/persistence was initially detected by follow-up US in eight, serum calcitonin analysis in two, and positron emission tomography-computed tomography (^18^F-labeled fludeoxyglucose) in one. All patients with structural recurrence/persistence showed suspicious US features on follow-up US. Final diagnosis of tumor recurrence/persistence was performed by US-guided fine-needle aspiration in six patients, and second-look surgery in five. Nine of the 11 patients with structural recurrence/persistence exhibited nodal recurrence/persistence, and two exhibited non-nodal recurrence/persistence. In this group, the mean period and session number of the follow-up US in which tumor recurrence/persistence was initially detected were 41.3 ± 39.3 months (range 6–128 months) and 2.6 ± 2.3 (range 1–8), respectively. Six of the 11 patients were diagnosed with tumor recurrence/persistence within 3 years of the initial surgery (6, 12, 21, 22, 25, and 33 months), and the remaining 4 were diagnosed at 50 (fifth), 62 (fourth session), 92 (seventh session), 98 (seventeenth session), and 128 (eighth session) months after the initial surgery. The structural recurrence/persistence group showed a mean serum calcitonin level of 662.5 ± 1634.7 pg/mL (range 19.2–5545.0 pg/mL).

Among the patient demographics variables and general characteristics of the primary tumor, tumor recurrence/persistence was significantly correlated with TNM stage and multiplicity/bilaterality of the tumor (Table 2). The primary MTCs in the recurrent/persistent group exhibited higher T and N stages than those of the non-recurrent group (both *p* < 0.001). Preoperative distant metastasis was only reported in 1 patient who showed tumor recurrence/persistence during the follow-up period. In the non-recurrence group, 56 patients (87.5%) exhibited solitary MTC in surgical specimen. However, there was a high prevalence of multiple and/or bilateral MTCs in the recurrence/persistence group compared with the non-recurrence group (*p* = 0.013). Factors such as age, sex of the patient, and the location and method of surgical excision of the tumor did not differ significantly between the recurrence/persistence and non-recurrence groups. There were statistically significant differences in mean interval and session number of postoperative follow-up US between the non-recurrence group (74.7 months and 6.4, respectively) and the tumor recurrence/persistence group (89.9 months and 8.7, respectively) (*p* = 0.013 and 0.005, respectively).

**Table 2:** Comparison of the factors related to tumor recurrence/persistence.

Items	Non-recurrence (*n* = 64)	Recurrence/persistence (*n* = 22)	*P*-value
Age (mean ± SD, years)	52.3 ± 12.6	53.5 ± 12.0	0.708
Sex			0.999
Female	39 (60.9)	14 (63.6)	
Male	25 (39.1)	8 (36.4)	
**Type of thyroid surgery**			0.755
Total thyroidectomy	57 (89.1)	21 (95.5)	
Hemithyroidectomy	6 (9.4)	1 (4.5)	
Subtotal thyroidectomy	1 (1.6)	0 (0)	
**Size of primary MTC (mm)**	15.9 ± 14.4	19.7 ± 11.1	0.263
**Location of primary MTC**			0.999
Right	44 (68.8)	15 (68.2)	
Left	19 (29.7)	7 (31.8)	
Isthmus	1 (1.6)	0 (0)	
**T stage**			<0.001
T1a	20 (31.3)	2 (9.1)	
T1b	25 (39.1)	4 (18.2)	
T2	8 (12.5)	2 (9.1)	
T3	11 (17.2)	14 (63.6)	
T4a	0 (0)	0 (0)	
**N stage**			<0.001
Nx (unknown)	3 (4.7)	0 (0)	
N0	36 (56.3)	2 (9.1)	
N1a	16 (25)	4 (18.2)	
N1b	9 (14.1)	16 (72.7)	
**M stage**			<0.001
Mx (unknown)	6 (9.4)	9 (40.9)	
M0	58 (90.6)	12 (54.5)	
M1	0 (0)	1 (4.5)	
**Multiplicity/bilaterality**			0.013
Solitary	56 (87.5)	14 (63.6)	
Satellite MTC in the ipsilateral lobe	2 (3.1)	4 (18.2)	
Satellite MTC in the contralateral lobe	0 (0)	1 (4.5)	
Satellite MTC in both lobes	6 (9.4)	3 (13.6)	
**Interval of the last follow-up US (mean ± SD, months)**	74.7 ± 24.2	89.9 ± 23.8	0.013
**Session number of the last follow-up US**	6.4 ± 2.8	8.7 ± 4.0	0.005

*Data presented in parentheses are percentage of each item*.

*MTC, medullary thyroid carcinoma; US, ultrasonography*.

In preoperative serologic tests, the serum calcitonin level was significantly higher in the tumor recurrence/persistence group (mean 4202.9 ± 15380.3 pg/mL, range 52.3–67633.0 pg/mL) than in the non-recurrence group (mean 277.4 ± 775.9 pg/mL, range 1.5–5790.0 pg/mL) (*p* = 0.042). In the last serologic test, there was a significant difference in the serum calcitonin levels of the two groups (*p* = 0.002). The tumor recurrence/persistence group showed significantly higher levels of serum calcitonin (mean 563.2 ± 1362.4 pg/mL, range 6.1–5545.0 pg/mL) than the non-recurrence group (mean 3.2 ± 2.2 pg/mL, range 1–9.3 pg/mL).

## Discussion

Many studies have investigated various prognostic factors in MTC patients (7–9, 23). Distant metastasis (known to be the main cause of mortality), advanced age, primary tumor stage, and nodal stage have been proposed as prognostic factors (7–9). As in these prior studies, TNM stage and multiplicity/bilaterality of the primary tumor were significantly correlated with tumor recurrence/persistence in our study. However, age and sex of the patient were not correlated with tumor recurrence/persistence in the present study, although they have been reported as prognostic factors in previous studies (8, 9, 23). The reason for this difference is unclear. However, the small sample size of MTC patients and tumor recurrence/persistence cases may be associated with discrepancy.

In the present study, the serum calcitonin levels detected in the preoperative and final serologic tests were compared between tumor recurrence/persistence and non-recurrence groups. Higher serum calcitonin levels are widely known to be predictive of MTC. Cohen et al. demonstrated that tumor size was significantly correlated with preoperative serum calcitonin level (24). Also, they reported that a preoperative serum calcitonin level <50 pg/mL was predictive of postoperative calcitonin normalization. Several studies have reported that postoperative calcitonin level is of predictive value with regard to disease progression and persistence (21, 25, 26). In our study, serum calcitonin levels in both preoperative and postoperative serologic tests were significantly correlated with tumor recurrence/persistence, as has been reported in previous studies (21, 25, 26).

In MTC patients, rates of structural recurrence ranging from <1 to 10.6% have been reported (21, 22). The rate of structural recurrence/persistence was 12.8% (11/86) in our study. Most of these (54.5%, 6/11) were detected within 3 years after the initial thyroid surgery. However, the remaining tumor recurrence/persistence cases were detected on follow-up US during 4–11 years after thyroid surgery. Of the 11 patients with structural recurrence/persistence, 8 had the initial detection of recurrence/persistence by follow-up US. Based on our study results, we recommend the following method of follow-up US for detecting tumor recurrence/persistence in MTC patients: (1) the follow-up US with annual interval is performed within 3 years after thyroid surgery and (2) the follow-up US with biennial interval is performed during 4–10 years after thyroid surgery. However, this method may be changeable depending on risk factors of tumor recurrence/persistence. For clarity, a large-scale study may be required.

In the present study, there were significant differences in mean interval and mean session number of follow-up US between the non-recurrence group and the tumor recurrence/persistence group. Namely, tumor recurrence/persistence group had longer periods of follow-up US, and more frequent sessions than the non-recurrence group. The reason for this may be associated with that recurrent/persistent patients had been diagnosed at a point in time during their follow-up, consequently they underwent more frequent follow-up US over a longer period of time than non-recurrence patients.

This study had several limitations. First, the sample size of the study was small. This can compromise the statistical interpretation of results. Second, retrospective analyses of medical records and US images were performed. This may have resulted in unavoidable selection bias. Third, we did not distinguish tumor recurrence from persistence. Finally, there were differences between the affiliated hospitals with regard to postoperative follow-up US protocols and serum calcitonin measurement. To overcome these limitations, a prospective study with a large patient population must be considered in the near future.

In conclusion, the tumor recurrence/persistence rate of MTC in the neck was 25.6% (22/86), the proportion of structural recurrence/persistence was 50% (11/22), and tumor recurrence/persistence was associated with advanced TNM stage and multiplicity/bilaterality. In the majority of patients with structural recurrence/persistence in the neck, tumor recurrence/persistence was detected during early follow-up US, within 3 years from the initial thyroid surgery. Thus, for detecting MTC recurrence/persistence in the neck, annual US surveillance may be sufficient within the first 3 years after thyroid surgery, but depending on the presence of relevant risk factors, annual or biannual US surveillance may be recommendable for 4–10 years after thyroid surgery.

## Ethics Statement

Informed consent was waived for all study participants due to the retrospective analysis, and the study design was approved by the appropriate ethics review boards (IRB 2016-0068). The data and materials in this manuscript have not been published or presented elsewhere in part or in entirety, and are not under consideration by another journal.

## Author Contributions

Concept and design: DK. Acquisition of data: all authors. Literature review: HA and DK. Analysis and interpretation of data: HB and DK. Manuscript writing: HA. Refinement of manuscript: all authors. Review of final manuscript: DK and YL.

## Conflict of Interest Statement

The authors declare that the research was conducted in the absence of any commercial or financial relationships that could be construed as a potential conflict of interest.
